# Breakthrough COVID-19 infections – Analyzing our experience

**DOI:** 10.12669/pjms.39.5.6724

**Published:** 2023

**Authors:** Aneela Hussain, Samreen Sarfaraz, Sabiha Anis, Quratulain Sheikh, Anum Rahim, Shameem Behram, Rabeea Shah, Owais Khan

**Affiliations:** 1 Dr. Aneela Hussain, MBBS, FCPS (Internal Medicine), FCPS (Infectious Diseases) Infectious Diseases Department, Indus Hospital & Health Network, Karachi, Pakistan; 2 Dr. Samreen Sarfaraz, MBBS, MRCP, FRCP Infectious Diseases Department, Indus Hospital & Health Network, Karachi, Pakistan; 3 Dr. Sabiha Anis, MBBS, MCPS (Clinical Pathology), FCPS (Immunology) Immunology Department, Indus Hospital & Health Network, Karachi, Pakistan; 4 Dr. Quratulain Shaikh, MBBS, FCPS (Internal Med.), MSc Epidemiology. & Biostatistics Infectious Diseases Department, Indus Hospital & Health Network, Karachi, Pakistan; 5 Dr. Anum Rahim, MBBS, MSc Epidemiology and Biostatistics, Indus Hospital Research Centre, Indus Hospital & Health Network, Karachi, Pakistan; 6 Dr. Shameem Behram, MBBS, FCPS (Internal Medicine), FCPS (Infectious Diseases) Infectious Diseases Department, Indus Hospital & Health Network, Karachi, Pakistan; 7 Dr. Rabeea Shah, MBBS, FCPS (Internal Medicine) Infectious Diseases Department, Indus Hospital & Health Network, Karachi, Pakistan; 8 Dr. Owais Khan, MBBS, FCPS (Internal Medicine) Infectious Diseases Department, Indus Hospital & Health Network, Karachi, Pakistan

**Keywords:** COVID-19, Breakthrough infections, Clinical characteristics, Ant-COVID-IgG antibodies

## Abstract

**Objective:**

There are many cases of post-vaccination COVID-19 globally. Also, literature on serum antibodies after vaccination is abundant. Our research focuses on breakthrough infections reported at our institution during the third wave of COVID-19.

**Methods:**

A total of 177 people recruited at the Indus Hospital Karachi between May to September 2021 with COVID-19 infection were divided into vaccinated, partially vaccinated, and unvaccinated cohorts. Furthermore, a subset of the vaccinated cohort was tested for anti-NP and anti-S antibodies.

**Results:**

There were 119 patients with breakthrough infection, however, 74% had mild symptoms. The antibodies against NP and S were found at a higher level in those who had a breakthrough infection in comparison to healthy vaccinated controls.

**Conclusion:**

Vaccination does not prevent disease but does confer some immunity causing less severe infection.

## INTRODUCTION

The COVID-19 pandemic has negatively impacted global health and economy. Mass vaccination drives continue using vaccines of varying platforms and efficacy in attempts to decrease transmission and severity. Many cases of breakthrough infections have been reported.1-3 These are defined as the detection of SARS-CoV-2 RNA in respiratory specimens of patients >14 days after vaccination.4 The CDC has noted 5800 breakthrough infections, mostly above 60 years, out of which one third were asymptomatic,470 severe and 74 fatal cases. 65% were women.5

There are many studies that show robust antibody response post-COVID-19 vaccination, most data being for mRNA vaccines.6 One study found that low anti-Spike antibody levels post-vaccination pose a high risk for breakthrough infection and these antibodies may wane earlier in AstraZeneca as compared to Pfizer.7 Another study found maximum antibody response with Pfizer, followed by AstraZeneca, Sputnik, and Sinopharm showing that individuals were at a higher risk for breakthrough infections who got the latter three vaccines, especially Sinopharm.8 In Pakistan, Sinopharm was the primary vaccine administered to most populations initially, we are seeing breakthrough infections mandating a study on breakthrough COVID-19 and antibody levels.

## METHODS

This study was conducted between 18^th^ May to 20^th^ September 2021 at the Indus Hospital Karachi under the Ethics committee approval number IHHN_IRB_2021_06_017 on 13^th^ July 2021. All Health care workers (HCWs) who tested positive for COVID-19 by a nasopharyngeal swab RT-PCR were considered eligible after informed consent. All symptomatic HCWs and those with exposure to COVID-19 patients, reported to the Infection Control department (ICD) of the hospital for COVID-19 nasopharyngeal RT-PCR. We classified positive cases into three categories: ‘breakthrough infection’ (at least two weeks had passed after completion of vaccine), ‘unvaccinated’ (COVID within one week of first dose of vaccine) and ‘partially vaccinated’ (tested positive after one week of first dose and the second dose was missed). Demographic and relevant clinical details were taken. Hospitalized COVID-19 breakthrough infection patients were also recruited after consent.

A subset of HCWs with breakthrough infections were registered for serum anti-COVID-IgG testing, where first sample was taken within a week of testing positive for COVID-19 via nasopharyngeal swab RT-PCR, and the second sample after one month. A control group comprising of healthy vaccinated individuals who never had COVID-19, was recruited for comparison. Blood samples for the control were drawn at 4-8 weeks after vaccination. Anti-COVID-NP-IgG and anti-COVID-S-IgG were tested using enzyme linked immunosorbent assay (ELISA) kits provided by AESKU, Germany. The antibody titers of <8 IU/ml was taken as negative, between 8 and 12 IU/ml as indeterminate and >12 IU/ml was considered as positive according to manufacturer’s instructions.

Data analysis was done using SPSS version 26.0. The normality of quantitative variables was assessed using Shapiro Wilk test. The normally distributed variables, the duration between symptoms and last day of vaccination was compared between the groups using students *t*-test. While for the non-normal variables such as age, duration of symptoms, total duration of illness and maximum ordinal score, Mann Whitney U test was applied. Chi-square test was applied to compare categorical variables, gender, type of vaccination and history of COVID-19. Other categorical variables were compared using fisher exact test. P-value of less than 0.05 was considered significant.

## RESULTS

In the study duration, 248 HCW infections were reported to ICD, of which 207 (83.5%) were breakthrough, 20 (8%) partially vaccinated and 21 (8.5%) unvaccinated respectively. All cases recovered without hospitalization. On the other hand, 750 COVID-19 patients were admitted during this time period, of which only 33 (4.4%) were breakthrough, 22 (2.9%) were partially vaccinated and 695 (92.7%) were unvaccinated. 200 (26.6%) of admitted patients expired, of which 8 (4%) were breakthrough, 4 (2%) were partially vaccinated and 188 (94%) were unvaccinated respectively.

A total of 206 participants were recruited; 177 were COVID-19 cases and 29 healthy controls. Among cases 119 were true breakthrough, 50 were partially vaccinated and eight were unvaccinated. Clinical characteristics of true breakthrough and partially vaccinated cases. Table-I The predominant vaccine administered in breakthrough cases was Sinopharm 74(62%) followed by Sinovac 32(27%) and CanSino BIO 13(11%) respectively. Although 80% of breakthrough cases were asymptomatic to mild symptomatic, moderate, severe, critical to fatal diseases were still observed in 5%, 5.8%, and 9.2% cases respectively. Eighteen point six percent of breakthrough cases were re-infections . Home isolation and symptomatic care were needed in 80.7% of breakthrough cases while 16.8% needed hospitalization (Table-I).

**Table-I:** Characteristics of true breakthrough and partially vaccinated cases

	True breakthrough: n (%)	Partial vaccination: n (%)
	119	50
Age	34.0years (28.0-45.0 years)	36.5 years (30.0-60.0years)
***Gender***		
Male	70(58.8)	34(68.0)
Female	49(41.2)	16(32.0)
Total	119 (100)	50
Occupation*		
A. Health care workers	86(72.2)	30(60)
Doctor	34(28.6)	3(6)
Nurse	8(6.7)	3(6)
Technician	10(8.4)	10(20)
Lab worker	6(5)	2(4)
Pharmacist	4(3.4)	2(4)
Housekeeping/security staff	9(7.6)	3(6)
Other Health care worker	15(12.6)	7(14)
B. Admitted patients (All were non hospital employees)	33(27.7) 119	20(40) 50
Type of vaccine*		
Sinopharm	74(62)	21(42)
Sinovac	32(27)	27(54)
CanSino BIO	13(11)119	2(4) 50

Onset of infection after vaccination (in days) **	*(n=111)*	*(n=39)*

Mean (SD)	92.5±47.2	51.8±34.2
***Comorbid***		
None*	91(76.5)	30(60.0)
Diabetes	11(9.2)	12(24.0)
Hypertension	21(17.6)	10(20.0)
IHD	3(2.5)	5(10.0)
Chronic Kidney Disease	1(0.8)	2(4.0)
Liver disease	1(0.8)	1(2.0)
Others	14(11.8)	6(12.0)
Use of immunosuppressive therapy	5 (4.2)	0
Previous history of COVID-19	22(18.6)	4(8.2)
***Duration of previous COVID-19***		
<1 year	12(54.5)	4(100)
>1 year	10(45.5)	0
***Severity of symptoms***		
Asymptomatic	7(5.8)	3(6)
Mild	88(73.9)	31(62)
Moderate	6(5.04)	2(4)
Severe	7(5.8)	7(14)
Critical	11(9.2)	7(14)
***Symptoms***		
Fever	116(97.4)	42(84)
Rhinorrhea	84(70.5)	5(10)
Cough	102(85.7)	22(44)
Lethargy	86(72.2)	7(14)
Body aches	93(78.2)	14 (28)
Shortness of breath	101(84.8)	19(38)
Diarrhea	85(71.4)	5(10)
***Outcomes***		
Patients’ Management		
Recovered with home isolation & symptomatic care	96(80.7)	30(60)
Received ED based remdesivir & steroids but not hospitalized	3(2.5)	0
Hospitalization	20(16.8)	20(40)
Required non-invasive ventilation	9(45)	6 (30)
Invasive ventilation	4(20)	1 (5)
Needed low flow oxygen	3 (15)	4 (20)
Needed high flow oxygen	4 (20)	3 (15)
Duration of hospitalization in days	(n=33)	(n=22)
Median (IQR)	5.0(3.0-9.3)	6.0(3.0-8.0)
***Survival status***		
Alive	111(93.3)	46(92)
Died	8(6.7)	4(8)
***Total duration of illness***		
Median (IQR)	7.0(5.0-10.0)	7.0(5.0-10.0)

COVID-19 IgG was estimated for 63 participants including 34 HCWs with breakthrough infections and 29 healthy vaccinated controls. Their baseline characteristics are described in Table-II. The cases were significantly younger by age than controls (*P=* 0.044). Gender, co-morbidity, and type of vaccines administered among cases and controls were not statistically significant. Sinopharm was received by 79.4% of breakthrough infection cases and 82.7% of controls.

**Table-II:** Baseline characteristics of breakthrough cases vs Controls (in whom anti-COVID-19-IgG was tested)

	Breakthrough	Controls	P-value
	34: n (%)	29: n (%)	
Age*	37.9(10.9 years)	44.6(13.2 years)	0.044
Gender*			
Male	20(58.8)	10(34.5)	0.077
Female	14(41.2)	19(65.5)	
Occupation*			0.401
Doctor	16(47)	19(65.5)	
Nurse	2(5.9)	0	
Technician	1(2.9)	0	
Lab Worker	1(2.9)	3(10.3)	
Other health care worker	14(41.2)	7(24.1)
***Type of vaccine***			0.494
Sinopharm	27(79.4)	24(82.7)	
Sinovac	4(11.8)	1(3.5)	
CanSino BIO	3(8.8)	4(13.7)	
***Comorbidity***			1.000
None	26(76.5)	23(79.3)	
Asthma	3(8.8)	3(10.3)	
Hypertension	2(5.9)	0	
IHD	0	2(6.8)	
Hypercholesteremia	2(5.9)	3(10.3)	
Others*^#^	3(8.8)	1(3.4)	
***Median anti-COVIDIgG with or without comorbidities***			
Anti-COVID-NP-IgG median (IU/ml) (min-max)			
Comorbidity-Present	100(1.9-100)	4.7(1.6-34.1)	0.000
Comorbidity-Absent	76(1-100)	10.5(1-100)	
***Anti-COVID-S-IgG median (IU/ml) (min-max)***			
Comorbidity-Present	82.5(95.4-100)	9.9(1-100)	0.030
Comorbidity-Absent	100(1-100)	50(1-100)	

© Chi-square test, £ Fisher Exact Test, ¥ Man-Whitney U test,

* p-value <0.05, *^#^=include valvular heart disease, prostatic cancer, CNS infection and Diabetes in one patient each.

No significant difference was found in the positivity rate of COVID-19 IgG antibodies between two groups, however, both anti-NP and anti-S antibodies coexisted in breakthrough patients at a higher rate while isolated anti-S antibodies was found in higher percentage of healthy controls. Median titers of both antiNP and antiS antibodies were significantly higher in breakthrough patients (Table-III). We then divided our patients and controls according to their post-vaccination period at which their samples were collected and found no difference in the positivity rates of both antibodies between groups except for anti-NPantibodies that were found higher in patients than controls when tested before four weeks post vaccination. However, median titers of these antibodies were significantly high in patients compared to controls at both time points.

**Table-III:** Comparison of anti-COVIDIgG between patients with breakthrough infection and vaccinated healthy controls.

	Breakthrough infections			Healthy controls			*P-values*		
Duration post-vaccination median (min-max)	4 months (0.7-9)			3.3 months (1.03-9.1)			0.619		
	Total (n=34); n (%)	≤4 months (n = 25) n (%)	>4-months (n =9) n (%)	Total (n = 29) n (%)	≤4 months (n = 19) n (%)	>4-months (n=10) n (%)	*P1^1^*	*P2^2^*	*P3^3^*
Anti COVID-IgG antibodies									
Anti-NP^4^ &-S^5^-IgG positive	24 (71)	21 (84)	3 (33)	14 (48)	10 (53)	3 (30)	0.799	0.099	0.486
Anti-NP^4^-IgG positive	2 (6)	1 (4)	1 (11)	0	0	0			
anti-S^5^-IgG positive	5 (15)	2 (8)	3 (33)	8 (28)	6 (32)	2 (20)			
anti-NP^4^ &-S^5^-IgG negative	3 (9)	1 (4)	2 (22)	7 (24)	2 (11)	5 (50)			
Anti-COVID-NP^4^-IgG antibodies									
Positive	26 (77)	22 (88)	4 (44)	14 (48)	10 (56)	3 (30)	0.087	0.055	0.384
Negative	6(18)	2 (8)	4 (44)	12 (41)	5 (28)	7 (70)			
Indeterminate	2 (6)	1 (3)	1 (11)	3 (10)	4 (17)	0			
Median (IU/ml) (min-max)	93.3(1.4-100)	100 (1.4-100)	8.9 (1.9-100)	10.0 (1-100)	12.9 (4.2-100)	2.8 (1-14.9)	0.000^6^	0.002^6^	
Anti-COVID-S^5^-IgG antibodies									
Positive	29 (85)	23 (92)	6 (67)	22 (76)	16 (89)	5 (50)	0.000^6^	0.450	0.675
Negative	4 (12)	1 (4)	3 (33)	7 (24)	2 (11)	5 (60)			
Indeterminate	1 (3)	1 (4)	0	0	0	0			
Median (IU/ml) (min-max)	98.2 (1-100)	100.0 (1-100)	83 (5-100)	48.5 (1-100)	58 (7-100)	24 (1-100)	0.032^6^	0.032^6^	

***Footnotes:***

1 = P-value between breakthrough infections (n=34)and Healthy controls (n=29),

2 = P-value between breakthrough infections (n=25 )and Healthy controls (n=19) less than or equal to 4 months post-vaccination,

3 =P-value between breakthrough infections (9) and Healthy controls (10) more than 4 months post-vaccination,

4 = nuclear capsid proteins,

5 =spike proteins,

* =significant P-value.

Amongst the 27 patients, re-tested at four to eight weeks, most of them found initially positive for anti-NP antibodies remained positive (18; 95%) while one became indeterminate. Of six samples that were initially tested negative, five turned positive at this time (83%). While of two indeterminate samples, one became positive and one negative on repeat testing (*P*=0.097). For anti-S antibodies, 23 samples were initially positive, three were negative and one indeterminate had same results on repeat testing (Table-III).

## DISCUSSION

It is a naive belief that previous COVID-infection or vaccination confers life-long immunity as SARS-CoV-2 is a non-monotypic virus. Immune response to COVID-19 is heterogeneous and influenced by several host characteristics causing a clinical spectrum ranging from asymptomatic to fatal disease. Similarly, immune protection after vaccination is determined by a complex interplay between host factors like age, gender, genetics, ethnicity, comorbidity, the predominant viral strain, immune evasion, inoculum, community transmission, the type of vaccine and post-vaccination period.^9^ All vaccines in current use are efficacious at limiting the disease rather than blocking infection.^10^ Thus vaccines help in overall reduction in the disease burden, preventing death, severity and hospitalizations (most data being available for mRNA vaccines) but mild and asymptomatic breakthrough COVID-19 infections continue to be reported globally. 11-13

We observed similar findings in our study where breakthrough cases had predominantly mild symptoms, needed symptomatic care, and only 16.8% required hospitalization. Unfortunately, 6.7% of breakthrough infections ultimately proved fatal, highlighting the fact that vaccination does not completely prevent death. Morbidity and mortality in patients hospitalized with severe disease were seen mainly in the unvaccinated cases.

The predominant circulating strain being reported globally and in Pakistan at the time of the study was the Delta variant, a highly transmissible variant causing severe disease with reported attenuated response from vaccination.^14^ The risk of severe breakthrough infection, however, remains low especially with mRNA vaccines like the BNT162b2 vaccine, while other vaccines being less effective.^14^,^15^ Breakthrough infection caused by Delta variant is comparable in transmission to that in unvaccinated individuals with similar viral burden in the upper respiratory tract.^16^ A decrement in vaccine efficacy has been reported after 12 weeks.^17^ The vaccine rollout in Pakistan has been mainly with the inactivated whole-virus vaccines Sinopharm, Sinovac and the Spike protein expressing adenovirus five vector based CanSino BIO, and robust literature on these vaccines is still unavailable. The overall reported efficacy from phase three trials for these three vaccines are 73%, 83.5% and 57.5% respectively, with CanSino BIO trial reporting 91.7% efficacy in prevention of severe disease.^18^,^19^ However, their performance against the Delta variant has not been described. The circulating Delta virus combined with types of vaccine used for the primary vaccination may be the prime reason for breakthrough infections among HCWs observed during the study period, who were at a high risk of exposure to the virus.

Ideally, a vigorous antibody response in any infection correlates with good outcome but till-date no antibody threshold has emerged to correlate the protection against COVID-19 breakthrough infections.^20^ Neutralizing antibodies against the spike protein and its receptor-binding domain have been reported as primary immune predictors of protection against symptomatic infection and increasing antibody levels correlate with higher vaccine efficacy.^21^,^22^ In Pakistan, neutralizing antibody titers are not available, and only anti-S or anti-NPIgG antibody titers are available commercially. Some correlation between neutralizing antibody titers and anti-S antibodies has been reported but its application to clinical care is uncertain.^23^

In this study, significantly high antibody titers for both anti-NP and anti-S antibodies were seen in breakthrough patients confirming previous study findings.^24^ The reason can be explained that antigen challenge has stimulated preformed memory B-cells as a result of vaccination or previous COVID-19 infection. This is supported by the fact that in healthy controls, anti-S antibody titers were found low and anti-NP antibodies were mostly absent. Another reason of high positivity and high antibody titers in breakthrough patients could be an aberrant immune response leading to symptomatic COVID-19 on one hand and early, uneventful recovery due to the stimulation of already activated effector cells and molecules of immune system secondary to vaccination. Presence of anti-NP antibodies in some of our healthy controls can be due to prior asymptomatic COVID-19 infection.

It has been reported that immunosuppression, comorbidity and old age result in a diminished immune response.^24^ In our cohort, the asthmatic patients on steroids confirm the pattern of more severe disease though the numbers are very low. Interestingly median age of patients was found low in breakthrough infections compared to controls.^24^

### Limitations

Our study has several limitations. Estimation of neutralizing antibodies and test for cell mediated immunity were not performed. Serum antibodies against COVID-19 were performed on only 63 patients, further splitted in cases and controls, a number too small to make an effective comparison. No immune correlates could be identified and repeat antibody testing could only be done in 27 patients; thus, no pattern of immunity overtime could be identified. Further, the variants/clades of SARS-CoV-2 were not determined.

## CONCLUSION

Our study found that breakthrough infections are usually mild which points towards some protection provided by vaccines. The anti-NP and anti-S titers were higher in infected people in comparison to healthy controls which could be a booster response to viral exposure. Further studies are needed to understand human immunological response to COVID-19.

### Authors Contribution:

**AH:** Conception of the idea, designing the study, literature search, data collection and compilation, write up and reviewing the article, responsible and accountable for accuracy and integrity of the article.

**SS and SA:** Conception of the idea, designing the study, data collection, write up and reviewing the article.

**QS:** Designing the study, data collection and write up.

**AR:** Data analysis, write up, data cleaning and management and reviewing the article.

**SB, RS, OK:** Data collection, compilation, reviewing the article.
